# Proof of concept for multiplex amplicon sequencing for mutation identification using the MinION nanopore sequencer

**DOI:** 10.1038/s41598-022-12613-7

**Published:** 2022-05-20

**Authors:** Whitney Whitford, Victoria Hawkins, Kriebashne S. Moodley, Matthew J. Grant, Klaus Lehnert, Russell G. Snell, Jessie C. Jacobsen

**Affiliations:** 1 School of Biological Sciences, The University of Auckland, Private Bag 92019, Auckland, 1142 New Zealand; 2 Centre for Brain Research, The University of Auckland, Auckland, New Zealand

**Keywords:** Genetics, Sequencing, DNA sequencing, Targeted resequencing

## Abstract

Rapid, cost-effective identification of genetic variants in small candidate genomic regions remains a challenge, particularly for less well equipped or lower throughput laboratories. The application of Oxford Nanopore Technologies’ MinION sequencer has the potential to fulfil this requirement. We demonstrate a proof of concept for a multiplexing assay that pools PCR amplicons for MinION sequencing to enable sequencing of multiple templates from multiple individuals, which could be applied to gene-targeted diagnostics. A combined strategy of barcoding and sample pooling was developed for simultaneous multiplex MinION sequencing of 100 PCR amplicons. The amplicons are family-specific, spanning a total of 30 loci in DNA isolated from 82 human neurodevelopmental cases and family members. The target regions were chosen for further interrogation because a potentially disease-causative variant had been identified in affected individuals following Illumina exome sequencing. The pooled MinION sequences were deconvoluted by aligning to custom references using the minimap2 aligner software. Our multiplexing approach produced an interpretable and expected sequence from 29 of the 30 targeted genetic loci. The sequence variant which was not correctly resolved in the MinION sequence was adjacent to a five nucleotide homopolymer. It is already known that homopolymers present a resolution problem with the MinION approach. Interestingly despite equimolar quantities of PCR amplicon pooled for sequencing, significant variation in the depth of coverage (127×–19,626×; mean = 8321×, std err = 452.99) was observed. We observed independent relationships between depth of coverage and target length, and depth of coverage and GC content. These relationships demonstrate biases of the MinION sequencer for longer templates and those with lower GC content. We demonstrate an efficient approach for variant discovery or confirmation from short DNA templates using the MinION sequencing device. With less than 130 × depth of coverage required for accurate genotyping, the methodology described here allows for rapid highly multiplexed targeted sequencing of large numbers of samples in a minimally equipped laboratory with a potential cost as much 200 × less than that from Sanger sequencing.

## Introduction

The rapid evolution of the study of genetic variation and the discovery of disease susceptibility and causal variants has led to a demand for methods for rapid variant discovery or accurate verification. Identifying a causative disease mutation can guide clinical management plans and lead to the identification of life-saving treatments^1,2^. The development of short read high throughput re-sequencing, particularly whole exome sequencing (WES) and whole genome sequencing (WGS) has accelerated this discovery process.

Decreasing cost has resulted in rapid adoption of these re-sequencing techniques and an increase in the rate of new pathogenic mutations discovery^3,4^. Trio sequencing (where the proband and their parents are sequenced) aids in variant analysis by determining the inheritance of putative causative variants. However, sequencing all members of a trio rather than just the proband is a significant extra cost. Sanger sequencing of selected PCR amplicons in close relatives such as parents is most often used to confirm inheritance of variants^2^. However, if there are numerous loci of interest in an individual or across a cohort, this can be a slow and costly exercise^5^.

As an alternative to single amplicon, sample by sample, Sanger sequencing, we considered that there may be efficiency gains to be made by multiplex sequencing using the Oxford Nanopore Technologies (ONT) MinION nanopore sequencer. This device is a low-cost USB-powered sequencer that returns sequencing results in real-time^6^. Due to the low cost and small size of the MinION, it is a viable, easy to operate tool for minimally equipped genetics labs. Sequencing and bioinformatics analysis for the MinION sequencer is possible using software that is both readily available and free of charge, on a laptop that meets minimum system requirements. The main limitation of MinION sequencing is the high nucleotide error rate^7^, currently between ~ 6 and 8%^8^. However, the high error rate can be mitigated by using multiple reads to establish a consensus following alignment or assembly.

The barcoding of PCR amplicons with barcodes available from ONT enables simultaneous sequencing of multiple samples, reducing the per-sample cost. The effectiveness of sequencing multiplex PCR amplicons from multiple individuals has been demonstrated in forensics^9,10^, heritable genetic disease^11,12^ and pharmacogenetic screening applications^13^. Notably, this approach played a significant role in tracking viral spread during the Zika^14^ and COVID-19^15–17^ outbreaks. A previous report by Liou et al. leveraged MinION sequencing as an alternative to Sanger sequencing for multilocus sequencing to strain type samples of *Staphylococcus aureus,* sequencing a pool of 672 PCR amplicons from the same sample^18^. This approach was assisted by a dual barcoding approach whereby barcodes (additional to the ONT native barcode) were incorporated into the PCR amplicons using primers with barcode sequence incorporated to the 5′ end of primers. This approach enabled the differentiation of sequences of the same target region from multiple samples (in this case, typing 96 bacteria in the same sequencing run).

In this report, we present proof of concept for a method to pool different amplicons under ONT native barcodes to simultaneously sequence a large number of amplicons, exceeding the number of barcodes used. Specific genetic loci, and therefore amplicons, were selected based on the clinical presentations for each family in the cohort. Only individuals within the appropriate pedigree were subject to PCR amplification and sequencing for that locus. In total, we sequenced 100 PCR amplicons from 82 participants pooled under ten barcodes, investigating sequence variance at 30 genetic loci. To our knowledge, this is the first report describing a method where diverse and sample-specific PCR amplicons can be pooled under the ONT native barcodes, allowing for high-throughput sequencing of any amplicon using the MinION.

## Materials and methods

### Samples

The study cohort consisted of 82 people comprised of 55 individuals with autism spectrum disorder who had been whole exome sequenced (Supplementary Methods), and 27 parents of the cases who had not undergone high-throughput sequencing. In total, 26 pedigrees were included consisting of 21 trios, three quads, and two quints. The genetic variants selected for further investigation were considered to be putative disease-causing mutations (Supplementary Methods). These variants consisted of 28 single nucleotide variants and two indels (a 1 bp deletion and a 2 bp insertion) (Supplementary Methods). DNA from the cohort was extracted from whole blood using the Qiagen Gentra Puregene Blood Kit (Hilden, Germany) or from saliva Oragene prepIT L2P saliva extraction kit (DNA GEnotek Inc, Ottawa, Canada) according to the manufacturer’s protocols.

DNA source is an important consideration for whole genome sequencing due to the presence of non-human DNA (primarily bacteria) extracted from saliva samples which can result in atypical alignments, and subsequent false positive variant calls, from whole genome sequencing reads^19^. However, it has been previously established that due to the specificity of targeted amplification of genetic regions prior to sequencing, saliva and blood-derived DNA display comparable purity, PCR amplification, and genotyping in targeted applications^20^. Therefore, we would not expect any anomalous variants to result from applying the assay presented here on mixed source DNA.

### Individual PCR

Amplicons incorporating each of the putative mutations ranged in size from 122 to 707 bp (variant location and PCR primers listed in Supplementary Table 1). Each PCR reaction mix consisted of 0.5 µM each of the forward and reverse primer, 1 × KAP2G Buffer A, 0.2 mM dNTP mix, and 0.5 units KAPA2G Robust DNA Polymerase, combined in a 25 µl reaction (Kapa Biosystems, Massachusetts, USA). The PCR cycling conditions were as follows: initial denaturation of 95 °C for 3 min, followed by 35 cycles of 95 °C for 15 s, 60 °C for 15 s, and 72 °C for 30 s, followed by a final 72 °C extension for 1 min.

The PCR amplicons were purified using Ampure XP magnetic beads (Beckman Coulter, California, USA) and quantified by Qubit Fluorometric Quantification Broad Range Assay (Invitrogen, California, USA). All PCR amplicons were Sanger sequenced, and the target variants identified for comparison.

### Strategy overview

**Figure 1:**
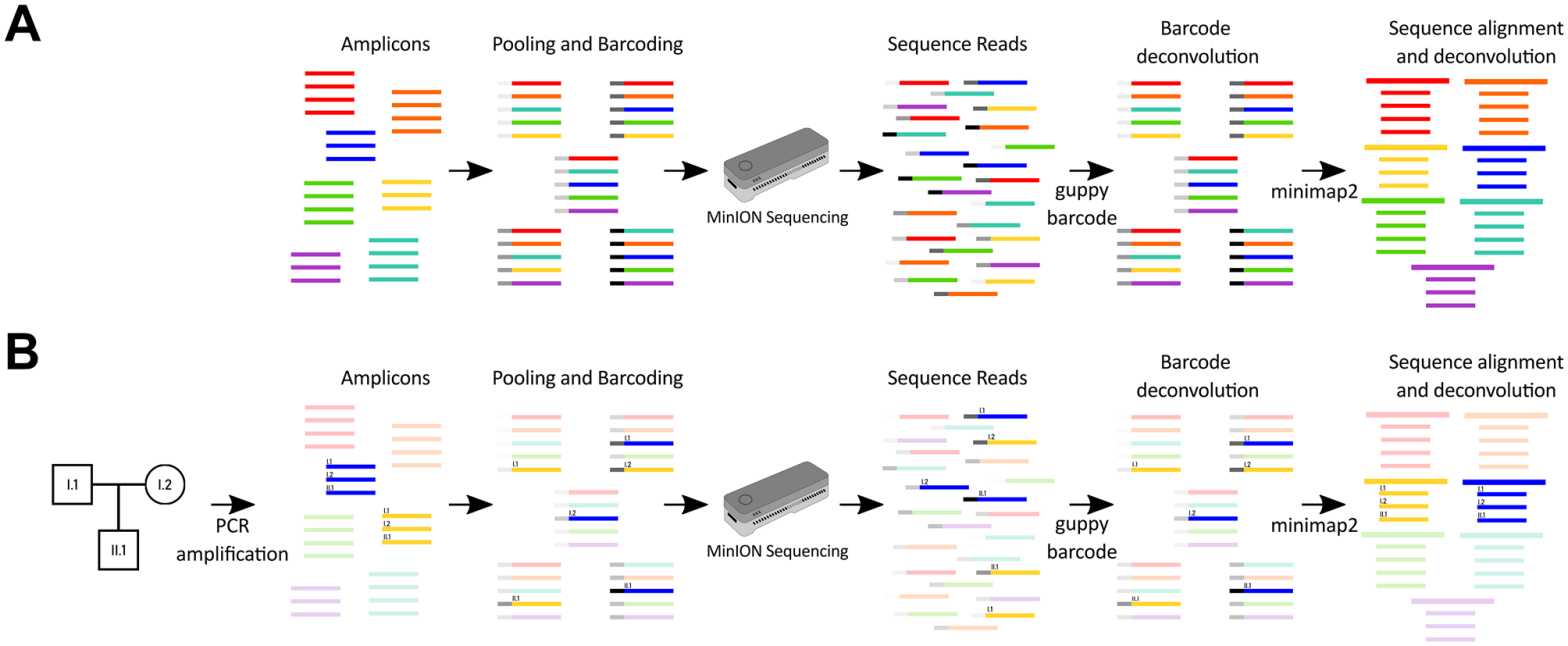
Representative strategy overview. (**A**) Amplicons from different target regions can be pooled and barcoded using ONT’s Native barcodes. The subsequent library is sequenced using the MinION sequencer. Each colour represents a different target amplicon. Each pool is barcoded with unique ONT Native barcodes, represented by distinct shades of grey. The resultant sequence reads then undergo two deconvolution steps: separation of reads according to barcode using guppy barcoder, followed by alignment of reads to the target region in a custom reference sequence with minimap2. Multiple individuals (such as multiple members of a pedigree) can be sequenced for the same target region as denoted by multiple lines for each PCR target (colour). Target amplicons are pooled, ensuring no pool contains more than one PCR product targeting the same region. The pools are then combined into a single library for sequencing using the MinION sequencer. The resultant reads are basecalled and assigned to the correct barcode by guppy. Finally, the barcoded sequence is aligned to a custom target reference by minimap2, allowing for the assignment of reads to the correct target amplicon and individual. (**B**) An example of how a single pedigree (trio) with two different target amplicons (coloured blue and yellow) could be sequenced using the strategy described in panel A. Following PCR amplification, the two PCR amplicons for the three members of the pedigree (I.1, I.2 and II.1) are pooled, sequenced, and deconvoluted as described in panel A. Other pedigrees are faded to focus on how a single pedigree can be tracked through the strategy.

Briefly, the strategy employed in this proposed method involves the pooling of unique PCR products, barcoding the pools using ONT’s Native Barcodes, sequencing the barcoded DNA using MinION sequencing, followed by two stages of deconvolution: separating sequence into the appropriate barcode using guppy barcode, and assigning reads to the correct sequence template by aligning to a custom target reference using minimap2. An overview of the process is presented in Fig. 1. The specific barcoding strategy used here is depicted in Supplementary Fig. 1.

### Sample pooling and barcoding

In order to analyse the MinION pooled sequence, a strategy for deconvoluting the sequence reads and assigning a read uniquely to a single sample of origin is required. Thus, we leveraged the step of sequence alignment to separate the PCR amplicons pooled under a single barcode. Each demultiplexed barcoded pooled sequence was aligned to a barcode-specific custom-generated reference—specifically, a fasta file with the sequence of each PCR amplicon target region pooled within a specific barcode. As the deconvolution relies upon the assignment of sequence reads to the correct target sequence, similarity between target sequences within the same reference could result in reads aligning to the wrong target. In order to ensure the correct assignment of reads to the corresponding target reference sequence, PCR amplicons pooled under a single barcode should not contain significant sequence similarity. Therefore, provided there is no similarity between target regions, the minimum number of barcodes is equal to the number of individuals for which the target region is being sequenced.

For convenience, the PCR amplicons in this experiment were randomly divided into ten pools (ten DNA templates in each pool) with no pool containing more than one PCR product targeting the same region (Supplementary Fig. S1). This ensured there was no similarity within pooled PCR amplicons within individual barcodes. Similarity was determined by BLAST Global Alignment^21^, where a fasta file of the target sequences for a single barcode was queried against itself using default parameters. For each barcode, each target region shared 0% similarity with all other targets.

0.16 pmol of each sample was added to each pool for a total of 1.6 pmol per pool. DNA templates within each pool were ligated to a specific barcode according to the 1D Native barcoding DNA (with EXP-NBD103 and SQK-LSK109) protocol (ONT).

### MinION library preparation

The barcoded pooled amplicon DNA was purified using Ampure XP magnetic beads. Each purified barcoded amplicon pool was quantified with Qubit, and pooled in equimolar quantities to generate the final library. The final 700 ng DNA library was prepared for MinION sequencing as in the 1D Native barcoding DNA (with EXP-NBD103 and SQK-LSK109) protocol (ONT).

### MinION sequencing data analysis

The MinION sequencer was run for 1 h 14 min, using a single new R9, 9.4 flowcell. Bases were called with guppy basecaller v6.0.1 (ONT) from the raw fast5 sequencing files from the MinION sequencer, followed by barcode demultiplexing also using guppy barcoder v6.0.1 (ONT) (with the barcoding kit (EXP-NBD104) specified), generating a fastq file for each barcode. Alternate basecalling was also performed using albacore v2.3.4 (ONT) and scrappie v1.4.0-0d933c7 (ONT), followed by barcode demultiplexing using guppy (as above). Pool demultiplexing and alignment was performed for all fastq files using minimap2.

Alignment and coverage statistics were calculated using samtools 1.10^22^.

Variants were called and genotyped from the aligned MinION sequencing reads using nanopolish v0.13.2 and then compared with the Sanger generated data^23^.

### Ethics approval and consent to participate

The genetic analysis and de-identified publication of variants for use cases was performed under the approval of the New Zealand Northern B Health and Disability Ethics Committee (12/NTB/59) in accordance with guidelines and regulations in the Ethical Guidelines for Observational Studies from the New Zealand National Ethics Advisory Committee. Parents and affected offspring provided written informed consent (with parents providing consent on behalf of their children, where appropriate).

## Results

### Read characteristics

A single sequencing run of the MinION produced 1,096,297 reads corresponding to approximately 11,000 reads per amplicon. Of the total reads, 988,051 (90.1%) passed the quality qscore of seven as determined by guppy, with a mean qscore of 9.9 and median qscore of 10.3. Guppy assigned the majority of reads (910,275) to the expected ONT 1–12 Native barcodes, with 94.32% of the correctly barcoded reads aligned to the reference by minimap2. The average sequence error rate was 2.94% for substitutions, 2.54% for deletions, and 1.69% for insertions as determined by Alfred^24^, consistent with expected error rates for the guppy basecaller^13,25^. Metrics of the run are detailed in Table 1.

**Table 1:** Metrics of the nanopore sequencing run.

Parameters	Metrics
Total yield	~ 632 million bp
Read length	13–112,160 bp (N50 = 587)
**Number of reads**	
Total	1,096,297
Pass	988,051
Reads mapped to reference	891,009
Average reads per amplicon (min–max)	8910.06 (138–20,896)
Average mean depth per amplicon (min–max)	8320.69 (127.53–19,626.4)
Mean qscore	9.9

### Genotype validation

Sequences were basecalled by guppy and aligned to the appropriate reference by minimap2. Nanopolish called 29/30 of the variants of interest (97/100 PCR amplicons). The genotypes called by nanopolish were in concordance with the previously generated Sanger sequence. The variant which could not be identified by nanopolish was an insertion of an AT at the junction of two adenosines and a homopolymer run of five guanine (ref: GGGGGAAT, alt: GGGGGAA**AT**T; Supplementary Table 1).

The ability to call the correct number of bases in homopolymer runs is a known limitation of the Oxford Nanopore sequencing technology^26,27^. Wick et al. reported different levels of success for homopolymer calls from various basecallers^26^. Therefore, two alternative basecalling algorithms, albacore v2.3.4 (ONT) and scrappie v1.4.0-0d933c7 (ONT) were run in an attempt to identify the AT insertion. Two additional alignments were generated by minimap2 from the sequences generated from albacore and scrappie, however, neither could call the correct number of bases in the guanine homopolymer run nor identify the homopolymer adjacent indel.

We also investigated whether other genetic loci (outside of the variants of interest) were discordant between MinION and Sanger sequence results to establish a more representative measure of the error rate for the method described here. Aside from the homopolymer adjacent variant already discussed, no further false negative calls were found. However, Nanopolish called 38 variants which were not supported by the corresponding Sanger sequence results. Twenty-seven of the 38 variants consisted of homopolymer sequences that nanopolish called as having shorter repeat lengths than their Sanger sequencing derived lengths. A further seven of the 38 discordant variants were found to be in low complexity repetitive regions. The accurate analysis of this type of sequence region has already been shown to be problematic using MinION sequencing^8^. The remaining four incorrectly called variants all had support from < 22% of the aligned reads, just above the 20% default minimum candidate frequency required from nanopolish. To investigate if altering variant detection thresholds prevent the detection of these false positives (particularly outside of low-complexity regions), we called the variants again using nanopolish with an increased minimum candidate frequency threshold of 0.25. This resulted in the removal of 12/27 of the variants within homopolymer runs, 5/7 of the variants in repetitive sequence, and all four of the non-low complexity variants. Therefore, we suggest a raised minimum candidate frequency threshold for diploid regions of 25% would reduce the rate of false positives while retaining the majority of true positive calls.

### Depth of coverage analysis

**Figure 2:**
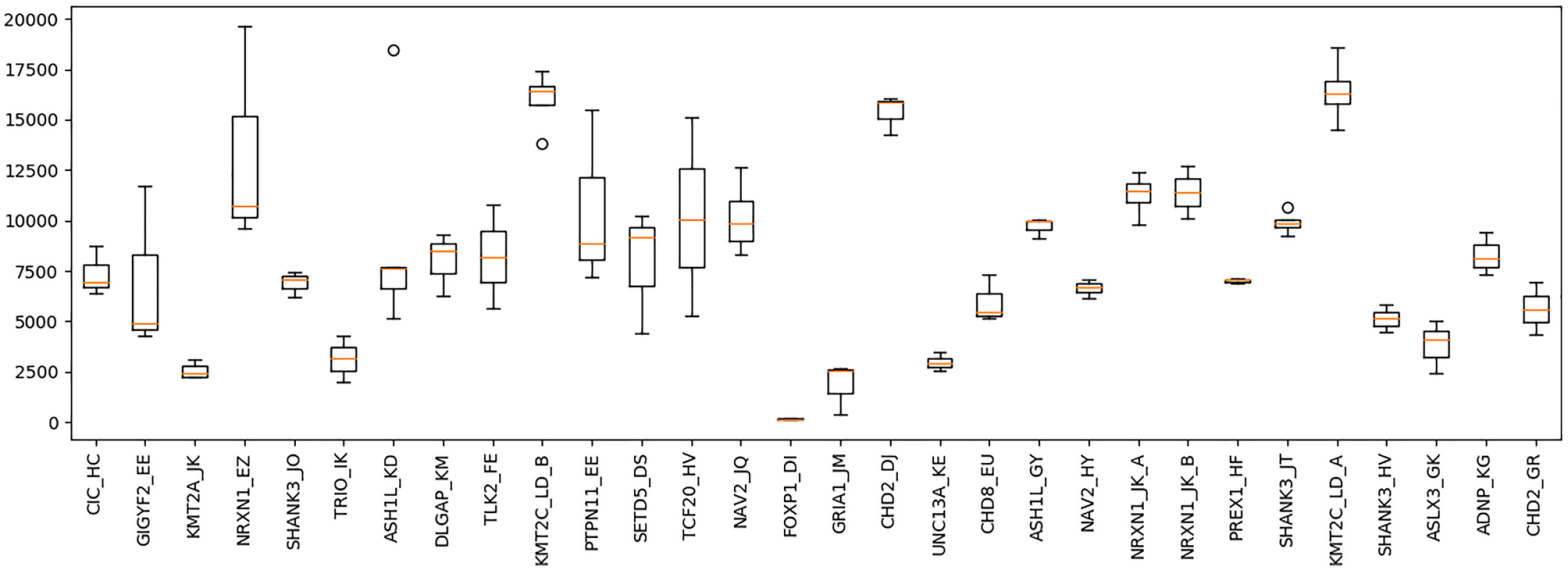
Depth of coverage of target regions. The name of each target region represents the gene name and the assigned pedigree ID. The average depth of sequence coverage varied significantly for different PCR amplicons, between 19,626.4 and 127.53 fold. The depth of coverage for PCR amplicons was significantly more uniform within target regions than between, with the average depth per target region varying significantly between 158× and 16418× [ANOVA: F(29, 70) = 10.05; P = 2.446 × 10^−15^]. Statistical outliers indicated by circles.

It was anticipated that pooling equimolar quantities for each PCR amplicon would result in comparable depth of coverage for each product when aligned to the respective reference. However, the average depth of sequence coverage for each amplicon varied widely between 19,626 and 127.53 fold (mean = 8320.69, std err = 452.99). The majority of variance was between target regions rather than between PCR amplicons of the same target region from different individuals (Fig. 2), with the average depth per target region varying significantly between 158× and 16418× [ANOVA: F(29, 70) = 10.05; P = 2.446 × 10^−15^].

**Figure 3:**
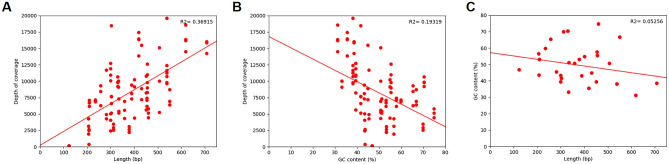
Alignment characteristics. (**A**) Each PCR amplicon is plotted (100 total), demonstrating a positive relationship between the length of the product and depth of coverage. (**B**) Each PCR amplicon is plotted (100 total), demonstrating a negative relationship between the GC content of the product and depth of coverage. (**C**) Length of amplicon compared to the GC content of each target region (30 total). The lack of significant relationship between these variables (R2 = 0.05256) indicates that each independently influences the depth of coverage of sequenced PCR amplicons.

There was a positive correlation between the nucleotide length of the PCR amplicon and the depth of coverage of the aligned sequence (Fig. 3A). Conversely, there was a negative correlation between PCR amplicon GC content and depth of coverage (Fig. 3B). While there was a negative correlation between PCR amplicon length and PCR amplicon GC content (Fig. 3C), the coefficient of determination is low (R^2^ = 0.0526). This suggests that they independently affect the depth of sequence coverage. The lack of relationship was ratified by an insignificant interaction term in a linear regression (P = 0.195).

## Discussion

The rapid uptake of genome re-sequencing following the human genome project has dramatically improved our understanding of human disease. The discovery of pathogenic and disease susceptibility variants has contributed to a shift from phenotype-driven diagnosis to genotype-driven diagnosis. While there has been a substantial decrease in sequencing cost in recent years, the limited funding available for research and molecular diagnostic facilities often does not allow for WGS or WES of all individuals recruited or referred for genetic analysis. Thus, sequencing of targeted DNA intervals remains a common low-cost alternative for applications such as mutational and inheritance confirmation^28^, forensics^9,10^ and targeted mutation screening for familial conditions^11^.

In large studies of multiple individuals, targeted sequencing by traditional methods such as Sanger sequencing is still relatively costly at approximately $7 USD per reaction^29,30^. Massively parallel sequencing platforms such as Illumina MiSeq offer a lower cost alternative to Sanger sequencing for targeted amplicon sequencing^31,32^. However, the initial capital expenditure for benchtop Illumina sequencers is outside of reach of most laboratories, with access to this technology remaining predominantly through a sequencing service provider. Due to the high accessibility and low cost-of-entry for both sequencer and bioinformatic analyses, we postulated that highly-multiplexed ONT MinION sequencing could also act as a high-throughput, low-cost alternative to Sanger sequencing. Subsequently, we have described a method to genotype loci in up to 100 PCR amplicons across 82 individuals simultaneously using Oxford Nanopore Technology’s MinION sequencer. The method described in this report is comparable in cost to Sanger sequencing ($6.24 USD per PCR amplicon^33^), with opportunities to reduce the cost further (as discussed below). An additional benefit of MinION sequencing for targeted re-sequencing is the rapid return of results with the process from library preparation to analysed results achievable within a day.

Oxford Nanopore Technologies sequencing incorporates native barcoding, facilitating the sequencing of multiple samples simultaneously. By pooling PCR amplicons targeting multiple regions within the same barcode, we have demonstrated the possibility to expand the utility of multiplexing with the MinION sequencing technology, reducing the per-amplicon cost of sequencing from $52 to $6.24 USD. The proof of concept method we described is not specific to the target regions that we used for the analyses presented here, nor specific to human variants. The user-specific genomic targets mean the method can be easily modified to determine the presence of any variant from any organism of interest by designing primers to the appropriate target region, provided the PCR amplicons to be pooled using a single barcode do not share significant similarity.

While this assay interrogated 100 PCR amplicons, the proposed assay could be expanded, further increasing cost-effectiveness. Provided the reads can be correctly demultiplexed, the limit to the number of amplicons that can be interrogated is determined by sufficient reads required for each amplicon and thus is a function of the yield of the flow cell and the length of the target regions of interest. Based on the error rate of 7.37% in this report and successful genotyping from samples with 128× and 164× coverage, we estimate that coverage of 100× should be adequate for establishing an accurate genotype in the majority of cases. This estimate is consistent with a previous report of 80× depth sufficient to generate consensus sequence^34^ and surpasses the minimum 20× depth of coverage from MinION sequencing to generate comparable sequence data to the Illumina MiSeq platform (as established by Radhakrishnan et al.^35^).

The reported maximum yield from a MinION single flow cell is 50 Gbp^33^. Therefore, with the metrics and amplicon size from this experiment, a theoretical 943,153 genetic loci could be accurately genotyped from a single flow cell with sufficient barcoding. However, this would require uniform depth of coverage for all amplicons and expending the full capacity of the flow cell over 72 h. Liou et al. also experienced an uneven distribution of reads with greater than four-fold difference in depth between the lowest depth and the mean^18^, and subsequently performed simulations to determine the maximum number of samples that could be genotyped in a single run. From a run of 6.3 million reads, consisting of 843 Mbp of sequence, with an average amplicon length of ~ 1300 bp, they determined a 95% accuracy in genotyping would be maintained with 7000 amplicons. Given the estimated minimum depth required for genotyping was consistent between our results and those from Liou et al., we expect that 7000 amplicons could also be used as an upper limit for the method that we have described here. For example, this method (with adequate barcoding) would allow for the screening 70 loci in 96 individuals, while only using 1.7% of the maximum yield of the flow cell.

One limitation of this method is the number of amplicons that can be sequenced targeting the same region of interest is constrained by the number of barcodes. For example, with the 10 barcodes used in this report, the same target genetic region could be sequenced in a maximum of 10 individuals. Expanding the number of amplicons targeting the same genetic region investigated in a single sequencing run can be supported by increasing the number of barcodes. This can either be through ONT native barcodes with a total of 96 available using the Native Barcoding Expansion 96 (EXP-NBD196) or through a dual barcode approach such as that used by Liou et al.^18^ and Srivathsan et al.^36^. The dual barcoding method applies barcoded PCR primers to incorporate barcodes into the products before pooling and the addition of secondary ONT native barcodes. Furthermore, given the small size of the PCR amplicons typically used for targeted sequencing, the assay could be optimised for the cheaper ‘Flongle’ MinION flow cells capable of generating up to 2.8 Gbp of sequence. The reported maximum yield from a single Flongle is more than threefold greater than the 843 Mbp used in the analysis by Liou et al. to estimate the maximum number of amplicons that could be genotyped in a single run^18^. As the number of amplicons sequenced in a single run increases, the cost per amplicon will reduce linearly. Therefore, utilisation of the Flongle for the method proposed here would reduce the cost of sequencing to $2.39 USD per amplicon if sequencing 100 amplicons, or as little as 3 cents if sequencing 7000 amplicons^37,38^.

Following deconvolution, 29 of 30 of the investigated genetic loci were successfully genotyped. Nanopolish identified 28 single nucleotide substitutions and one single base pair insertion from basecalls by guppy. The variant that was not identified during variant calling by any of the algorithms tested here is an insertion of adenosine and thymine at the junction of two adenosines and a homopolymer run of five guanines. We also identified 27 further instances where homopolymer runs were called the incorrect length, and seven variants in repetitive regions. The difficulty of basecalling MinION sequence surrounding stretches of low complexity is a well-reported limitation of the technology^8,9,36,39^.

Nanopore sequencing utilises nanoscale pores, for which the ionic current passing through the pore changes as DNA/RNA passes through. This change in current is used to determine the bases present in the pore and thus the nucleotide sequence of the template^27^. Consequently, a repeatedly reported limitation of the technology is the difficulty to correctly identify the length of homopolymer runs greater than the k-mer of the nucleotides determining the signal from the pore as these extended homopolymer runs do not result in a change in the current through the pore^27^. For the flowcells used in this experiment (R9, 9.4), the signal is primarily determined from the three central nucleotides within the pore^40^, thus accurately determining the length of a homopolymer greater than as few as four nucleotides is technically challenging. Similar to the increase in raw read accuracy that has been achieved with updates in chemistry and software tools^27^, we anticipate improvements to homopolymer calls. Particularly, ONT reports better homopolymer identification with R10, 10.3 pores released in January 2020^41^.

Oikonomopoulos et al. reported that the proportion of MinION reads very closely mirrors the number of template molecules in the library^39^. Therefore, we presumed including equimolar quantities of PCR amplicons would result in an equal depth of coverage for each product. However, we observed significant variance between target regions, with less variance between PCR amplicons from the same target. Therefore, inherent differences between the amplicons resulted in differences in read depth. Specifically, within the bounds of amplicons investigated here, MinION sequencing has a bias for longer amplicon length and lower GC content.

There are mixed reports of GC content and length bias in MinION sequencing. Oikonomopoulos et al. did not observe any length or GC content bias from the pooled library^39^. However, Li et al. directly compared the reads generated by MinION and Pacbio Sequel and Ion Torrent PGM and observed a significantly reduced number of reads with > 45% GC content from MinION relative to the other technologies^42^. Notably, there was a negative relationship between read counts and GC content > 40%. Both Li et al. and Karlsson et al. reported a normal distribution of the proportion of reads centred around ~ 7 kb^42,43^. This distribution replicates the positive relationship observed in this report if limited to the 122–707 bp window investigated here.

The lack of relationship between target sequence length and GC content indicated that the two variables independently affect depth of coverage. Therefore, pooling equimolar quantities of PCR amplicons may not be the best approach to achieve equal depth of coverage for all templates required for accurate variant identification while maximising the use of the flow cell. With a significantly larger number of sequencing runs targeting additional varied template sequences, a regression model incorporating depth of coverage, length, GC content and molarity may reveal the relationship between these variables and determine the optimum quantity of products to be pooled. Such a model will optimise the depth of coverage for each PCR amplicon, thus allowing for screening of the maximum number of amplicons in a single sequencing run. At this stage, the greatest impact on depth of coverage is template size. Therefore, when designing an assay, we recommend minimising the size difference between amplicons. MinION sequencing has been demonstrated to be suitable for routine diagnostics for both genetic variant screening^11,12^ and the identification of pathogens^44,45^. With an assay designed to reduce the variation of depth of coverage between targets, the method presented here could be further implemented in clinical diagnostics to reduce sequencing costs compared to Sanger sequencing.

## Conclusion

Oxford Nanopore Technology’s MinION sequencer allows for rapid and cost-effective development of variant discovery assays. In the proof of concept reported here, genotypes of targeted genetic loci were called with a 97% accuracy, the only exception being an insertion adjacent to a homopolymer run. The difficulty to correctly call the number of bases in a homopolymer run is a known limitation of the technology, which will likely be mitigated through improvements in chemistry and flow cells by ONT and the development of superior basecalling software. At this point, however, we recommend avoiding including variants in low complexity regions for interrogation using the method presented here. Depth of coverage does not scale with the number of molecules of DNA pooled. However, the depth achieved in this experiment was sufficient for successful variant calling in 29/30 of the genetic variants of interest. Therefore, ONT MinION has the potential to be used as a time and cost-effective alternative to Sanger sequencing for large-scale variant identification from any short DNA template with adequate barcoding and read depth.

## Supplementary Information

Supplementary Information.
