# Supplementary material for: Proof of concept for multiplex amplicon sequencing for mutation identification using the MinION nanopore sequencer
Source: Sci Rep. 2022 May 20;12:8572. doi: 10.1038/s41598-022-12613-7 (PMC9122479; doi:10.1038/s41598-022-12613-7)
Supplement: Supplementary file 1 — Supplementary Information. [file 41598_2022_12613_MOESM1_ESM.docx]

**Supplementary methods**

**DNA sequencing and alignment**

Whole exome sequencing was performed using the SureSelect XT Human All Exon v6 (Agilent, Santa Clara, CA, USA) and Illumina Novaseq 6000 by Macrogen (Seoul, Rep. of Korea) to obtain 59 million 100bp paired-end reads for each individual. Reads were mapped to the human GRCh37.p13 reference using bwa (v 0.7.4)^1^, duplicates marked with Picard (v 1.96)^2^ followed by indel realignment and base quality score recalibration using the Genome Analysis Toolkit (GATK, v 3.4-46). SNP and indel variants were identified using GATK HaplotypeCaller, and GenotypeGVCFs as described in Van der Auwera et al^3^.

**Candidate variant identification**

Genetic variants for this report were selected from a study focussing on the identification of causative genetic variants for individuals diagnosed with autism spectrum disorder (ASD). Variants were therefore prioritised if they resided in genes expressed in the brain^4^, had previously been reported as causative in ASD or neurodevelopmental disorders^5^, and by SFARI score (a scoring system based on the strength of evidence supporting the role of the gene to ASD risk^6^). Additionally, variants were filtered to prioritise those observed at a minor allele frequency of less than 0.1% in the 1000 Genomes^7^, ExAC^8^, genome aggregation database (gnomAD^8^), or our in-house dataset.

**Supplementary Table 1.** Amplicons Sequenced

| **Gene target and pedigree** | **Primers** | **Product size (bp)** | **GC content (%)** | **Variant** | **Individuals sequenced by minION** | **WES genotype; allele depth** | **MinION genotype; strand support** |
| --- | --- | --- | --- | --- | --- | --- | --- |
| CIC_HC | Primer F  5’ GTTGAAGATCCGTGAGGTGC 3’  Primer R  5’ TCACACGCTCCAGGTTATGT 3’ | 551 | 66.79 | ENST00000575354.2:c.4718C>T | II.1^†^  I.1  I.2 | 0/1; 11,12 | 0/1; 6501,4667,2106,3161  0/1; 4237,3375,3810,3237  0/0‡ |
| GIGYF2_EE | Primer F  5’ TCAGTCCATTTGAGTTTGCGG 3’  Primer R  5’ TCCTCTAAGCACCATTCGGG 3’ | 282 | 45.39 | ENST00000409451.3:c.817C>T | II.1^†^  I.1  I.2 | 0/1; 28,39 | 0/1; 1138,1062,1094,1194  0/0‡  0/1; 1264,1306,1223,1329 |
| PTPN11_EE | Primer F  5’ TCCTGACTTCTGCCACTTCGT 3’  Primer R  5’ CAAAAGGAGAGCGTATCCAAGAGG 3’ | 431 | 44.78 | ENST00000351677.2:c.1492C>T | II.1^†^  I.1  I.2 | 0/1; 20,24 | 0/1; 3516,4430,3289,4741  0/0‡  0/1; 1370,2417,1196,2517 |
| NRXN1_EZ | Primer F  5’ CATGTGAAGGGAGACCGTGT 3’  Primer R  5’ AGCGCGTGGTGAAAGATATTG 3’ | 538 | 38.10 | ENST00000404971.1:c.3071A>G | II.1^†^  I.1  I.2 | 0/1; 33,25 | 0/1; 2400,2596,2442,2557  0/1; 4897,5517,4846,5007  0/0‡ |
| SHANK3_JO | Primer F  5’ CAGGTGAGACCTGAGCGTG 3’  Primer R  5’ CAACACCAAATACCCCTCGC 3’ | 454 | 57.71 | ENST00000262795.3:c.898C>T | II.1^†^  I.1  I.2 | 0/1; 60,50 | 0/1; 1358,1822,1509,1786  0/0‡  0/1; 1819,2097,1841,1986 |
| TRIO_IK | Primer F  5’ GGTCCTATCAATCTGTCGGGG 3’  Primer R  5’ TCAGGGCCCTTCCAGGTAAT 3’ | 205 | 56.59 | ENST00000344204.4:c.8066T>C | II.1^†^  I.1  I.2 | 0/1; 62,48 | 0/1; 1011,580,1071,571  0/0‡  0/1; 691,270,765,280 |
| ASH1L_KD | Primer F  5’ TGACCTATGACCAACGTTCAAGT 3’  Primer R  5’ TCAAAGCATGAAAAGCAGCCTC 3’ | 303 | 41.58 | ENST00000392403.3:c.1731G>C | II.1^†^  II.2^†^  II.3^†^  I.1  I.2 | 0/1; 31,29  0/1; 37,28  0/1; 47,25 | 0/1; 2043,1446,1899,1545  0/1; 2566,1570,2334,1573  0/1; 1839,952,1585,994  0/1; 5749,3970,5479,3990  0/0‡ |
| DLGAP_KM | Primer F  5’ ACACTCGTCCTTCAGCTCTTG 3’  Primer R  5’ CCATGAAAGGGCTATCAGGCA 3’ | 257 | 65.37 | ENST00000315677.3:c.85C>T | II.1^†^  I.1  I.2 | 0/1; 20,15 | 0/1; 1839,952,1585,994  0/0‡  0/1; 2566,1570,2334,1573 |
| TLK2_FE | Primer F  5’ GAGGACTCTCCCTGAGTATCCA 3’  Primer R  5’ TAATGATTTGGGGAGAAAGTCTGC 3’ | 450 | 39.33 | ENST00000346027.5:c.1784C>G | II.1^†^  I.1  I.2 | 0/1; 22,21 | 0/1; 1817,2477,1828,2385  0/0‡  0/0‡ |
| KMT2C_LD_A | Primer F  5’ CCCACTTTATTGAAAGACTACAGGT 3’  Primer R  5’ AATCTTTCTTGTGAGGTCTAGTTGT 3’ | 619 | 31.18 | ENST00000262189.6:c.1759_1769del | II.1^†^  II.2^†^  I.1  I.2 | 0/0‡  0/1; 41,24 | 0/0‡  0/1; 4860,4752,4944,4743  0/0‡  0/0‡ |
| KMT2C_LD_B | Primer F  5’ AATGGTGAGTCAGAGTATCCCA 3’  Primer R  5’ AGTTATCGCTTAAAGCAGTTGAATA 3’ | 419 | 35.56 | ENST00000262189.6:c.467C>T | II.1^†^  II.2^†^  I.1  I.2 | 0/1; 28,32  0/1; 30,37 | 0/1; 4275,4541,3784,4199  0/1; 4450,4549,4324,4586  0/1; 4218,4524,3921,4226  0/0‡ |
| SETD5_DS | Primer F  5’ GGGACTTGTTCGCGTCCTTAT 3’  Primer R  5’ TCTGAGGTTGGCGAGTCTGA 3’ | 401 | 54.86 | ENST00000402198.1:c.3929C>T | II.1^†^  I.1  I.2 | 0/1; 43,58 | 0/1; 1334,967,1353,949  0/1; 3227,2221,3193,2148  0/0‡ |
| TCF20_HV | Primer F  5’ CAGTCGCTTTTCTGGTACCCC 3’  Primer R  5’ AATGCACAGGCTTATGGAACAC 3’ | 507 | 50.69 | ENST00000359486.3:c.1105A>G | II.1^†^  I.1  I.2 | 0/1; 79,71 | 0/1; 1331,1405,1347,1391  0/0‡  0/1; 4233,4076,3676,3717 |
| NAV2_JQ | Primer F  5’ TAGCACCTCGAACTGTCTGC 3’  Primer R  5’ GAGGCTCTGCATCATACCCA 3’ | 455 | 55.38 | ENST00000396087.3:c.7261G>A | II.1^†^  II.2^†^  II.3^†^  I.1  I.2 | 0/1; 65,47  0/1; 72,61  0/1; 55,53 | 0/1; 2512,2279,1771,2112  0/1; 2668,2484,1892,2385  0/1; 3517,3415,2887,3363  0/0‡  0/1; 2849,2648,2051,2717 |
| FOXP1_DI | Primer F  5’ CTGAGAAAGCTTACCTTCCACG 3’  Primer R  5’ TCACAGGCCATTCTCGAATCT 3’ | 122 | 46.7 | ENST00000491238.1:c.1472A>G | II.1^†^  I.1  I.2 | 0/1; 40,36 | 0/1; 28,22,27,15  0/1; 39,27,32,30  0/0‡ |
| GRIA1_JM | Primer F  5’ CTTTGGTCCGGGAAGAAGTT 3’  Primer R  5’ ATTCATAGGGACTGAAGCGGC 3’ | 207 | 43.49 | ENST00000518783.1:c.1568G>A | II.1^†^  I.1  I.2 | 0/1; 28,12 | 0/1; 103,86,87,95  0/0‡  0/0‡ |
| **CHD2_DJ** | **Primer F**  **5’ AGGCTCTTGCCAAAGGAACA 3’**  **Primer R**  **5’ CCAGTGTAGGAAGGTTGGGG 3’** | **707** | **38.61** | **ENST00000394196.4:c.2423_2424insAT** | **II.1^†^**  **I.1**  **I.2** | **0/1; 49,31** | **0/0‡**  **0/0‡**  **0/0‡** |
| UNC13A_KE | Primer F  5’ ATCTTGGTTCAGCACCGGG 3’  Primer R  5’ GAGTATTGCAGGGAGGCGTT 3’ | 312 | 69.87 | ENST00000428389.2:c.205G>A | II.1^†^  I.1  I.2 | 0/1; 16,18 | 0/1; 1400,311,1600,317  0/1; 1194,336,1261,296  0/0‡ |
| CHD8_EU | Primer F  5’ CACAATGCCAGCCGATCTTC 3’  Primer R  5’ TCCGCACTTTTGCTCGACT 3’ | 334 | 51.20 | ENST00000399982.2:c.5665C>T | II.1^†^  I.1  I.2 | 0/1; 34,36 | 0/1; 2528,1045,1137,991  0/1; 2114,978,1348,918  0/0‡ |
| ASH1L_GY | Primer F  5’ TATCACTGGTGTGCTTTACTTCCT 3’  Primer R  5’ GTTTCACTTGCCAGCATTTTTGC 3’ | 332 | 33.13 | ENST00000392403.3:c.7061T>G | II.1^†^  I.1  I.2 | 0/1; 25,25 | 0/1; 2186,2986,2091,2259  0/1; 2473,3247,2218,2512  0/0‡ |
| NAV2_HY | Primer F  5’ GACAAGTGTGTCTGCTTCGG 3’  Primer R  5’ AGCAGGTATTGCGTGGAAGG 3’ | 209 | 53.11 | ENST00000396087.3:c.2153G>A | II.1^†^  I.1  I.2 | 0/1; 84,73 | 0/1; 1318,1878,1331,1769  0/1; 1384,2264,1424,2222  0/0‡ |
| NRXN1_JK_A | Primer F  5’ CATGTGAAGGGAGACCGTGT 3’  Primer R  5’ AGCGCGTGGTGAAAGATATTG 3’ | 538 | 38.10 | ENST00000404971.1:c.3113G>A | Proband^†^  Sibling1^†^  Mother  Father | 0/0‡  0/1; 91,8^*^ | 0/0‡  0/0‡  0/0‡  0/0‡ |
| NRXN1_JK_B | Primer F  5’ ATGTGTTGATTGCCTTGCTTTGA 3’  Primer R  5’ GGTAGTGGAGGCCAAGTTGTAA 3’ | 300 | 39.33 | ENST00000404971.1:c.780A>T | Proband^†^  Sibling1^†^  Mother  Father | 0/1; 24,22  0/0‡ | 0/1; 2738,2479,2778,2521  0/0‡  0/1; 2940,2746,2916,2759  0/0‡ |
| KMT2A_JK | Primer F  5’ ACGTGGTGGACTCTAGTCAGA 3’  Primer R  5’ GCTGTTTGAGACATCAGTGCT 3’ | 378 | 53.17 | ENST00000534358.1:c.3974G>A | Proband^†^  Sibling^†^  Mother  Father | 0/1; 28,35  0/0^‡^ | 0/1; 371,1082,429,971  0/0‡  0/0‡  0/1; 351,894,350,776 |
| PREX1_HF | Primer F  5’ CCTTGGACTGGCTATCCCCT 3’  Primer R  5’ ACTTTGGGTTCCTCCTGTCA 3’ | 234 | 59.83 | ENST00000371941.3:c.3727C>T | II.1^†^  I.1  I.2 | 0/1; 104,66 | 0/1; 1787,1647,1927,1621  0/1; 1917,1752,1807,1722  0/0‡ |
| SHANK3_JT | Primer F  5’ AGTCACCCGAGGACAAGAAGT 3’  Primer R  5’ CCTCATCGCTGGACGACAG 3’ | 330 | 70.30 | ENST00000262795.3:c.3700G>A | Proband^†^  Sibling1^†^  Mother  Father | 0/1; 53,33  0/1; 42,25 | 0/1; 3437,2357,1380,2452  0/1; 3926,2702,1598,2858  0/0‡  0/1; 3620,2615,1475,2484 |
| SHANK3_HV | Primer F  5’ CCGGGTGGCCTCGACT 3’  Primer R  5’ GTACATCCACAAACAGGGGTC 3’ | 460 | 74.78 | ENST00000262795.3:c.3227C>G | II.1^†^  I.1  I.2 | 0/1; 40,27 | 0/1; 1236,1800,1303,1769  0/1; 833,1445,959,1477  0/0‡ |
| ASLX3_GK | Primer F  5’ TCCTAATGTCTGTTGACAGTGCAAA 3’  Primer R  5’ TTGGGGGAATCAAGACAGAGCTA 3’ | 300 | 43.33 | ENST00000269197.5:c.3889C>T | II.1^†^  I.1  I.2 | 0/1; 29,32 | 0/1; 1829,1004,1636,844  0/0‡  0/1; 928,405,851,389 |
| ADNP_KG | Primer F  5’ GCGGCCATCTTTTCCACATC 3’  Primer R  5’ TCTTCTCTCTCATCGGGCCA 3’ | 389 | 43.19 | ENST00000396029.3:c.1490A>G | II.1^†^  I.1  I.2 | 0/1; 32,41 | 0/1; 1213,2803,1131,3364  0/0‡  0/0‡ |
| CHD2_GR | Primer F  5’ TGCAGTCATCAGATCATTCTTTCT 3’  Primer R  5’ TCATGTATACGTCACTTTCTCCCT 3’ | 359 | 50.70 | ENST00000394196.4:c.5032C>T | II.1^†^  I.1  I.2 | 0/1; 91,68 | 0/1; 1817,1612,1807,1996  0/0‡  0/1; 1050,1113,1046,1317 |

Amplicon names represent the gene for which the locus of interest is located followed by the assigned pedigree ID

† Individuals for which whole exome sequence was available

*Variant determined to be homozygous reference by Sanger sequencing

‡Variants not called by variant caller are indicated by 0/0

Standard annotations of 0 for the reference allele, 1 for the first alternate, 0/0 for homozygous refence, and 0/1 for heterozygous variants.

The bolded row represents the variant that was unable to be correctly genotyped from aligned MinION sequence

**Figure S1. Amplicon Barcode Matrix.** Amplicons sequenced (gene target variant, pedigree and individual) demonstrating barcode allocation. A unique colour designates each target genetic loci for ease of interpretation.

**References**

1. Li, H. & Durbin, R. Fast and accurate short read alignment with Burrows-Wheeler transform. *Bioinformatics* **25**, 1754–1760 (2009).

2. BroadInstitute. Picard Tools - By Broad Institute. (2016). Available at: http://broadinstitute.github.io/picard/. (Accessed: 3rd September 2018)

3. Van der Auwera, G. A. *et al.* From FastQ Data to High-Confidence Variant Calls: The Genome Analysis Toolkit Best Practices Pipeline. in *Current Protocols in Bioinformatics* 11.10.1-11.10.33 (John Wiley & Sons, Inc., 2013). doi:10.1002/0471250953.bi1110s43

4. GTEx Consortium. The Genotype-Tissue Expression (GTEx) pilot analysis: multitissue gene regulation in humans. *Science (80-. ).* **348**, 648–660 (2015).

5. McKusick-Nathans Institute of Genetic Medicine, Johns Hopkins University (Baltimore, M. Online Mendelian Inheritance in Man, OMIM®. Available at: https://www.omim.org/. (Accessed: 4th March 2020)

6. Abrahams, B. S. *et al.* SFARI Gene 2.0: a community-driven knowledgebase for the autism spectrum disorders (ASDs). *Mol. Autism* **4**, 36 (2013).

7. The 1000 Genomes Project Consortium. A global reference for human genetic variation. *Nature* **526**, 68–74 (2015).

8. Lek, M. *et al.* Analysis of protein-coding genetic variation in 60,706 humans. *Nature* **536**, 285–291 (2016).
